# Neurofilament Light Chain and Cognitive Dysfunction in Patients Undergoing Hemodialysis: A Cross-Sectional Exploratory Study

**DOI:** 10.34067/KID.0000001162

**Published:** 2026-02-20

**Authors:** Kazuhiko Kato, Akio Nakashima, Shunichiro Shinagawa, Arisa Kobayashi, Ichiro Ohkido, Mitsuyoshi Urashima, Takashi Yokoo

**Affiliations:** 1 Division of Nephrology and Hypertension, Department of Internal Medicine, The Jikei University School of Medicine, Tokyo, Japan; 2 Division of Molecular Epidemiology, The Jikei University School of Medicine, Tokyo, Japan; 3 Department of Psychiatry, The Jikei University School of Medicine, Tokyo, Japan; 4 Human Health Sciences, Kyoto University Graduate School of Medicine, Kyoto, Japan

**Keywords:** dementia, hemodialysis, mineral metabolism

## Abstract

**Key Points:**

- Blood biomarkers related to amyloid *β* and neurofilament light chain are used to predict cognitive decline in the general population.
- Evidence for their usefulness is lacking in patients receiving hemodialysis despite their high risk of cognitive impairment.
- Neurofilament light chain levels were negatively associated with cognitive function, whereas the amyloid *β* ratio was not.

**Background:**

Cognitive dysfunction is frequently observed in patients undergoing hemodialysis in clinical settings; however, its pathophysiology remains uncertain. In recent years, noninvasive blood biomarkers have garnered increasing attention in predicting Alzheimer's disease development. Within the general population, neurofilament light chain (NfL) levels have been used to prognosticate changes in cognitive function alongside the amyloid *β* (A*β*) 1–42/1–40 ratio. Nevertheless, this has not been studied in patients with CKD, particularly those undergoing hemodialysis.

**Methods:**

This exploratory cross-sectional study investigated the association of the serum A*β*1–42/1–40 ratio and NfL levels with cognitive function assessed using the Montreal Cognitive Assessment (MoCA) and Mini-Mental State Examination (MMSE), in patients undergoing hemodialysis.

**Results:**

We included 384 patients with a median age of 74 (interquartile range [IQR], 70–80) years. The median (IQR) MoCA and MMSE scores were 25 (22–26) and 28 (26–29), respectively. The median (IQR) A*β*1–42/1–40 ratio and NfL level were 0.04 (0.03–0.06) and 196.2 pg/ml (146.5–262.2), respectively. The multivariate linear regression analysis indicated a weak negative association between the log-transformed NfL levels and cognitive function, after adjusting for confounding factors including age (*β* coefficient [95% confidence interval], −0.98 [−1.81 to −0.15]; *P* = 0.021 for MoCA and −0.66 [−1.32 to −0.01]; *P* = 0.046 for MMSE). However, the A*β*1–42/1–40 ratio was not associated with cognitive function.

**Conclusions:**

Our exploratory analysis identified a weak negative association between serum NfL levels and cognitive function in patients undergoing hemodialysis.

## Visual Abstract

**Figure:**
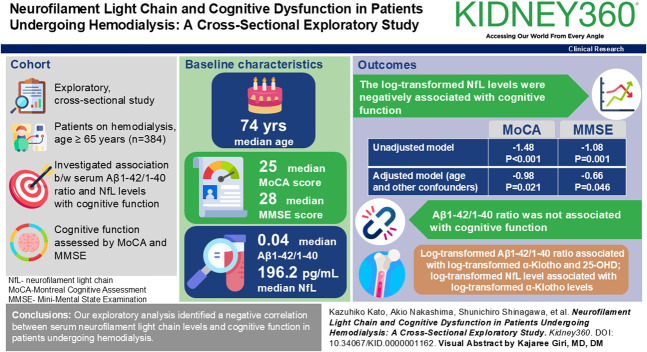

## Introduction

Cognitive dysfunction is a frequent clinical observation in patients undergoing hemodialysis^1^; however, its pathophysiology remains uncertain. Notably, Alzheimer's disease, a leading cause of cognitive dysfunction, is difficult to diagnose through clinical examinations. Moreover, current modalities, including cerebrospinal fluid examination and positron emission tomography, are invasive and costly. Consequently, biomarkers that improve diagnostic accuracy assume a prominent role in research and clinical practice.^2^ However, developing easily accessible biomarkers for use in primary care settings remains an unresolved concern.^3,4^

Blood biomarkers are being increasingly recognized as a novel approach with the potential to address this concern. Among these biomarkers, the amyloid *β* (A*β*) 1–42/1–40 ratio, p-tau181, and neurofilament light chain (NfL) hold promise for clinical application and are being actively investigated.^4–6^ However, their validation in diverse population-based cohorts remains incomplete.^7^ Notably, data for patients undergoing hemodialysis, who are at a high risk of cognitive decline, are lacking.^8^ Recent studies have focused on the A*β*1–42/1–40 ratio and tau protein,^9^ and NfL has been inadequately assessed owing to limited sample sizes in previous studies, thus hindering a comprehensive evaluation of its utility.^10,11^

NfL levels may increase independently of or in parallel with changes in A*β* levels. The underlying mechanisms of cognitive impairment in patients undergoing hemodialysis are multifactorial, involving both vascular and neurodegenerative components. Blood NfL levels increase because of axonal damage, regardless of the cause, especially in rapidly progressive neurodegenerative conditions.^6^ Thus, blood NfL levels may be a useful marker for detecting neuronal damage in patients undergoing hemodialysis. Despite this, few studies have simultaneously examined the relationship between blood NfL and A*β* levels and cognitive function in this population.

Furthermore, CKD-mineral and bone disorder (CKD-MBD) has been reported to be associated with cognitive impairment.^12,13^ Given the systemic effects of CKD-MBD, including its contribution to vascular calcification and potential effect on cerebral microcirculation,^14^ it may also influence pathways related to neurodegeneration and A*β* deposition. Consequently, it may be linked to neurodegeneration and A*β* accumulation; however, these relationships are yet to be elucidated.

Given this background, cerebrospinal fluid analysis and amyloid/tau positron emission tomography imaging are often impractical in older adults receiving hemodialysis, and the establishment of easily accessible blood biomarkers has major clinical implications. Therefore, determining whether blood biomarkers reflect cognitive vulnerability in this high-risk population may inform early identification strategies and support future clinical decision making.

Therefore, the aim of this exploratory study was to investigate the associations between NfL and A*β* levels and cognitive function in patients undergoing hemodialysis, as well as the potential relationships between CKD-MBD–related factors and blood biomarkers associated with Alzheimer's disease and neurodegeneration.

## Methods

### Ethical Approval

All procedures were performed in accordance with the ethical standards of the relevant institutional and national research committees and those of the 1964 Declaration of Helsinki and its later amendments or comparable ethical standards. The Institutional Review Board of the Jikei University School of Medicine approved this study (approval no.: 33-183 [10800]). Written informed consent was obtained from all individual participants included in this study.

### Study Population

This cross-sectional study included patients 65 years or older undergoing hemodialysis at seven hemodialysis centers who were not diagnosed with or treated for dementia. Patients with medical conditions that could acutely or chronically impair cognition were excluded. Specifically, we excluded individuals with active or untreated conditions known to affect cognitive function (such as delirium, acute psychiatric episodes, or severe electrolyte disturbances), and those with acute medical illnesses requiring ongoing or recent treatment at the time of assessment, including pneumonia, acute heart failure, acute infections, or other decompensated systemic disorders. In addition, patients who had been hospitalized for any acute medical condition within the preceding 3 months were not eligible for enrollment. In accordance with the study design, we also excluded patients who had undergone hemodialysis for fewer than 3 months, those receiving fewer than three hemodialysis sessions per week, and those for whom blood biomarker measurements could not be obtained. Patients were consecutively recruited from seven dialysis centers in Tokyo and Chiba, Japan, between November 2021 and November 2022, and all eligible patients were enrolled consecutively to minimize selection bias.

### Clinical and Laboratory Data Collection

Information regarding the patients' demographics, comorbidities, and current medications was recorded at the time of enrollment. Albumin (g/dl), calcium (mg/dl), phosphate (mg/dl), magnesium (mg/dl), and intact parathyroid hormone (PTH, pg/ml) levels were assessed using standard commercial assays before a hemodialysis session at the beginning of the week. Single-pool urea clearance was measured. All blood samples were centrifuged for 10 minutes and divided into tubes maintained at −80°C until analysis. Serum NfL levels^15,16^ were measured using an ELISA kit provided by UmanDiagnostics AB (Umeå, Sweden). Serum A*β*1–42 (High-Sensitive, no. 296–64401) and A*β*1–40 (no. 298–64601) levels were measured using an ELISA kit provided by FUJIFILM Wako Pure Chemical Corporation (Osaka, Japan). In addition, the A*β*1–42/1–40 ratio was calculated by dividing the A*β*1–42 level by the A*β*1–40 level. Serum 25-hydroxyvitamin D (25-OHD) levels (ng/ml) were measured using an electrochemiluminescence immunoassay kit (SRL Inc., Tokyo, Japan). Serum intact fibroblast growth factor-23 (FGF-23) and soluble *α*-Klotho levels were measured using ELISA kits provided by KAINOS Laboratories Inc. (Tokyo, Japan) and Immuno-Biological Laboratories Co., Ltd. (Gunma, Japan). All assays for each biomarker were performed using kits from the same manufacturing lot to minimize interassay variability. In addition, a survey was administered to procure information regarding the patients' educational level, smoking history, and exercise habits. The subjective sleep quality was evaluated using the Pittsburgh Sleep Quality Index.^17^

### Cognitive Function Assessment

Global cognitive function was evaluated using the Japanese versions of the Montreal Cognitive Assessment (MoCA)^18,19^ and Mini-Mental State Examination (MMSE).^20^ To minimize measurement bias, one trained investigator administered both assessments to all participants. Patients were assessed during hemodialysis, while they were sitting or lying on a hemodialysis bed at a time when they had no hypotension.

### Primary and Secondary Outcomes

The primary outcomes were the associations of the serum NfL level and A*β*1–42/1–40 ratio (blood biomarkers) with cognitive function. The secondary outcomes were the associations of these biomarkers with CKD-MBD–related factors (*α*-Klotho, FGF-23, 25-OHD, and magnesium levels).

### Statistical Analyses

Data are presented as the mean±SD or median with interquartile range (IQR). Data were log-transformed using the natural logarithm for the A*β*1–42/1–40 ratio and NfL, intact PTH, FGF-23, and soluble *α*-Klotho levels. First, the association of various blood biomarkers with cognitive function was evaluated by determining the Spearman rank correlation coefficient and illustrated in scatter plots and regression lines. A multivariate linear regression analysis examined the association between blood biomarkers and cognitive function. The disjunctive cause criterion was used as the covariate selection method, and variables that were causes of exposures, outcomes, or both, according to previous studies, were selected as confounders.^21^ Sex and age were adjusted for in model 1. In model 2, we adjusted for lifestyle factors (body mass index, first-degree family history of dementia, exercise habits, smoking history, Pittsburgh Sleep Quality Index score, and education level [college degree or higher or not]) as well as the factors adjusted for in model 1. In model 3, we adjusted for possible confounding comorbidities (diabetes, cardiovascular disease, cerebral hemorrhage, stroke, brain tumor, hearing loss, psychiatric illness, and head injury) and the factors adjusted for in model 2. In model 4, we adjusted for medications affecting cognitive function (benzodiazepines, antiepileptic drugs, histamine H1 receptor antagonists, and histamine H2 receptor antagonists), including the factors adjusted for in model 3. We ensured that none of the variables exhibited multicollinearity. Thereafter, the association of CKD-MBD–related factors (*α*-Klotho, FGF-23, 25-OHD, and magnesium levels) with blood biomarkers was evaluated by determining the Spearman rank correlation coefficient and analyzing them using multivariate linear regression analysis. For this analysis, we designed a new multivariate analytical model because the assumed confounding factors differed from those in the analysis examining the association between blood biomarkers and cognitive function. Sex and age were adjusted for in model 1. In model 2, possible confounding comorbidities (body mass index and history of diabetes, cardiovascular disease, cerebral hemorrhage, stroke, and head injury) were adjusted for, in addition to the factors adjusted for in model 1. In model 3, CKD-MBD–related factors (calcium, phosphate, and log-transformed intact PTH levels; urea clearance; and administration of calcimimetics, phosphate binders, and active vitamin D analogs) were adjusted for, in addition to the factors adjusted for in model 2. All tests performed in this study were two-sided, with values of *P* < 0.05 indicating statistical significance. A complete case analysis was performed when any data were found to be missing. Given the exploratory nature of the study, no adjustment for multiplicity was applied, and no formal sample size calculation was conducted. All statistical analyses were performed using Stata version 15.1 (StataCorp LLC, College Station, TX).

## Results

### Patient Characteristics

This study initially included 403 patients 65 years or older undergoing hemodialysis. During screening, patients were excluded if they had medical conditions that could acutely or chronically impair cognition (*n*=10), underwent hemodialysis fewer than three times per week (*n*=1), or had unavailable blood biomarker measurements (*n*=8). Table 1 presents the characteristics of the 384 patients included in the final analysis. The median (IQR) patient age was 74 (70–80) years. The median (IQR) MoCA and MMSE scores were 25 (22–26) and 28 (26–29), respectively. The median (IQR) A*β*1–42/1–40 ratio and NfL levels were 0.04 (0.03–0.06) and 196.2 (146.5–262.2) pg/ml, respectively. The median (IQR) dialysis vintage and body mass index were 87 (36–169) months and 21.6 (19.6–24.2) kg/m^2^, respectively. The mean (±SD) albumin level was 3.6 (±0.3) g/dl. The median (IQR) serum 25-OHD, intact FGF-23, and soluble *α*-Klotho levels were 14.1 (10–19.6), 1925 (608–4771), and 381 (301–516) pg/ml, respectively. The amount of missing data were small, and the number of missing observations for each variable is presented in Table 1.

**Table 1:** Patient characteristics (*n*=384)

Variable	Value	Missing, *n* (%)
Age, yr	74 (70–80)	0 (0)
Sex (male), *n* (%)	259 (67.5)	0 (0)
**Social and lifestyle factors**		
Body mass index, kg/m^2^	21.6 (19.6–24.2)	0 (0)
Family history of dementia, *n* (%)	49 (13.2)	14 (4)
Exercise habits, *n* (%)	158 (42.6)	13 (3)
Smoking history, *n* (%)	229 (64.7)	30 (8)
Pittsburgh Sleep Quality Index score	6 (4–8)	19 (5)
Education level, college degree or higher, *n* (%)	155 (40.7)	3 (1)
**Comorbidities**		
Dialysis vintage, mo	87 (36–169)	0 (0)
Diabetes mellitus, *n* (%)	163 (42.5)	0 (0)
Cardiovascular disease, *n* (%)	132 (34.4)	0 (0)
Cerebral hemorrhage, *n* (%)	19 (5)	0 (0)
Stroke, *n* (%)	43 (11.2)	0 (0)
Brain tumor, *n* (%)	3 (0.8)	0 (0)
Hearing loss, *n* (%)	92 (24.4)	7 (2)
Psychiatric illness, *n* (%)	14 (3.7)	10 (3)
Head injury, *n* (%)	30 (8)	8 (2)
**Treatment, *n* (%)**		
Benzodiazepines	74 (19.3)	0 (0)
Antiepileptic drugs	10 (2.6)	0 (0)
Histamine H1 receptor antagonists	62 (16.2)	0 (0)
Histamine H2 receptor antagonists	11 (2.9)	0 (0)
Calcimimetics	205 (53.4)	0 (0)
Phosphate binders	250 (65.1)	0 (0)
Active vitamin D analogs	287 (74.7)	0 (0)
**Laboratory test measurements**		
Kt/V	1.51 (1.3–1.7)	18 (5)
Albumin level, g/dl	3.6±0.3	0 (0)
Calcium level, mg/dl	8.3±0.6	0 (0)
Phosphate level, mg/dl	5.2 (4.4–5.9)	0 (0)
Intact PTH level, pg/ml	157 (94–239)	0 (0)
25-OHD level, ng/ml	14.1 (10–19.6)	0 (0)
Intact FGF-23 level, pg/ml	1925 (608–4771)	0 (0)
Soluble *α*-Klotho level, pg/ml	381 (301–516)	0 (0)
Magnesium level, mg/dl	2.4±0.3	0 (0)
**Blood biomarkers**		
A*β* 1–42 level, pmol/L	2.8 (1.8–4.3)	0 (0)
A*β* 1–40 level, pmol/L	73.8 (48.4–92.7)	0 (0)
A*β* 1–42/1–40 ratio	0.04 (0.03–0.06)	0 (0)
NfL level, pg/ml	196.2 (146.5–262.2)	0 (0)
**Cognitive function**		
MoCA score	25 (22–26)	0 (0)
MMSE score	28 (26–29)	0 (0)

Continuous variables are presented as means (±SDs) or medians (interquartile ranges). Categorical variables are shown as numbers of patients (%). 25-OHD, 25-hydroxyvitamin D; A*β*, amyloid *β*; FGF-23, fibroblast growth factor-23; Kt/V, urea clearance; MMSE, Mini-Mental State Examination; MoCA, Montreal Cognitive Assessment; NfL, neurofilament light chain; PTH, parathyroid hormone.

### Association between Blood Biomarkers and Cognitive Function

**Figure 1:**
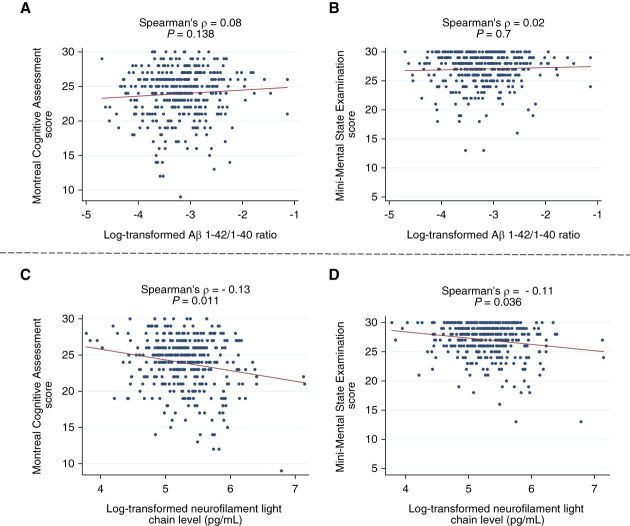
**Correlations between blood biomarkers and cognitive function.** (A) MoCA score and log-transformed A*β*1–42/1–40 ratio. (B) MMSE score and log-transformed A*β*1–42/1–40 ratio. (C) MoCA score and log-transformed NfL level. (D) MMSE score and log-transformed NfL level. A*β*, amyloid *β*; MMSE, Mini-Mental State Examination; MoCA, Montreal Cognitive Assessment; NfL, neurofilament light chain.

As shown in Figure 1, NfL levels exhibited a weak inverse correlation with cognitive function (Spearman rank correlation coefficient [ρ]: −0.13 and *P* = 0.011 for MoCA; −0.11 and *P* = 0.036 for MMSE). By contrast, the A*β*1–42/1–40 ratio was not correlated with cognitive function (ρ=0.08 and *P* = 0.138 for MoCA; ρ=0.02 and *P* = 0.7 for MMSE). In addition, linear regression analysis indicated a weak association between the log-transformed NfL level and MoCA (*β* coefficient [95% confidence interval (CI)], −1.48 [−2.25 to −0.7] and *P* < 0.001 in the unadjusted model; −0.98 [−1.81 to −0.15] and *P* = 0.021 in model 4) and MMSE (*β* coefficient [95% CI], −1.08 [−1.69 to −0.46] and *P* = 0.001 in the unadjusted model; −0.66 [−1.32 to −0.01] and *P* = 0.046 in model 4) scores (Table 2). However, the A*β*1–42/1–40 ratio was not associated with these scores.

**Table 2:** Univariate and multivariate linear regression analyses of blood biomarker levels in relation to Montreal Cognitive Assessment and Mini-Mental State Examination scores

Exposure and Model	MoCA Score		MMSE Score	
	*β* (95% CI)	*P* Value	*β* (95% CI)	*P* Value
**Log-transformed A*β* 1–42/1–40 ratio**				
Unadjusted	0.44 (−0.19 to 1.07)	0.174	0.16 (−0.31 to 0.7)	0.446
Model 1	0.37 (−0.24 to 0.98)	0.23	0.17 (−0.32 to 0.65)	0.5
Model 2	0.3 (−0.35 to 0.94)	0.366	0.05 (−0.45 to 0.56)	0.845
Model 3	0.38 (−0.27 to 1.03)	0.254	0.1 (−0.41 to 0.61)	0.707
Model 4	0.43 (−0.22 to 1.09)	0.194	0.13 (−0.38 to 0.65)	0.609
**Log-transformed NfL level**				
Unadjusted	−1.48 (−2.25 to −0.7)	<0.001	−1.08 (−1.69 to −0.46)	0.001
Model 1	−0.99 (−1.75 to −0.22)	0.012	−0.69 (−1.3 to −0.08)	0.027
Model 2	−0.9 (−1.72 to −0.08)	0.031	−0.7 (−1.34 to −0.06)	0.031
Model 3	−0.96 (−1.78 to −0.14)	0.022	−0.66 (−1.3 to −0.01)	0.047
Model 4	−0.98 (−1.81 to −0.15)	0.021	−0.66 (−1.32 to −0.01)	0.046

Model 1: adjusted for sex and age.

Model 2: adjusted for body mass index, family history of dementia, exercise habits, smoking history, Pittsburgh Sleep Quality Index score, and education level (college degree or higher) in addition to the factors adjusted for in model 1.

Model 3: adjusted for a history of diabetes, cardiovascular disease, cerebral hemorrhage, stroke, brain tumor, hearing loss, psychiatric illness, and head injury in addition to the factors adjusted for in model 2.

Model 4: adjusted for the oral administration of benzodiazepines, antiepileptic drugs, histamine H1 receptor antagonists, and histamine H2 receptor antagonists in addition to the factors adjusted for in model 3. A*β*, amyloid *β*; CI, confidence interval; MMSE, Mini-Mental State Examination; MoCA, Montreal Cognitive Assessment; NfL, neurofilament light chain.

### Association between CKD-MBD–Related Factors and Blood Biomarkers

As presented in Table 3, the A*β*1–42/1–40 ratio exhibited a weak correlation with the *α*-Klotho and 25-OHD levels (ρ=0.19 and *P* < 0.001 for *α*-Klotho; −0.2 and *P* < 0.001 for 25-OHD). In addition, the NfL level exhibited a weak correlation with the *α*-Klotho level (ρ=0.15 and *P* = 0.005). The multivariate linear regression analysis revealed weak associations of the log-transformed A*β*1–42/1–40 ratio with the log-transformed *α*-Klotho and 25-OHD levels and of the log-transformed NfL level with the log-transformed *α*-Klotho level (Table 4), similar to the results presented in Table 3. Moreover, the log-transformed intact FGF-23 level was weakly associated with the NfL level in one multivariate model (95% CI, 0.05 [0.01 to 0.09] and *P* = 0.009 in model 3).

**Table 3:** Correlations between blood biomarkers and CKD-mineral and bone disorder–related factors

Exposure	Log-Transformed A*β*1–42/1–40 Ratio		Log-Transformed NfL Level	
	Spearman Rank Correlation Coefficient	*P* Value	Spearman Rank Correlation Coefficient	*P* Value
Log-transformed soluble *α*-Klotho level, pg/ml	0.19	<0.001	0.15	0.005
Log-transformed intact FGF-23 level, pg/ml	0.05	0.371	−0.03	0.517
25-OHD level, ng/ml	−0.2	<0.001	−0.03	0.6
Magnesium level, mg/dl	−0.08	0.121	−0.03	0.551

25-OHD, 25-hydroxyvitamin D; A*β*, amyloid *β*; FGF-23, fibroblast growth factor-23; NfL, neurofilament light chain.

**Table 4:** Univariate and multivariate linear regression analyses of CKD-mineral and bone disorder–related factor levels in relation to blood biomarker levels

Exposure and Model	Log-Transformed A*β* 1–42/1–40 Ratio		Log-Transformed NfL Level	
	*β* (95% CI)	*P* Value	*β* (95% CI)	*P* Value
**Log-transformed soluble *α*-Klotho level, pg/ml**				
Unadjusted	0.18 (0.06 to 0.3)	0.004	0.19 (0.09 to 0.29)	<0.001
Model 1	0.17 (0.05 to 0.29)	0.006	0.2 (0.1 to 0.3)	<0.001
Model 2	0.17 (0.04 to 0.29)	0.008	0.22 (0.12 to 0.32)	<0.001
Model 3	0.14 (0.02 to 0.27)	0.027	0.21 (0.11 to 0.31)	<0.001
**Log-transformed intact FGF-23 level, pg/ml**				
Unadjusted	0.01 (−0.03 to 0.05)	0.717	−0.01 (−0.04 to 0.03)	0.651
Model 1	0.01 (−0.03 to 0.05)	0.568	0.01 (−0.03 to 0.03)	0.928
Model 2	0.01 (−0.03 to 0.05)	0.657	0.01 (−0.02 to 0.05)	0.438
Model 3	0.01 (−0.04 to 0.06)	0.649	0.05 (0.01 to 0.09)	0.009
**25-OHD level, ng/ml**				
Unadjusted	−0.01 (−0.02 to −0.01)	0.001	0.01 (−0.01 to 0.01)	0.614
Model 1	−0.01 (−0.02 to −0.01)	0.003	0.01 (−0.01 to 0.01)	0.901
Model 2	−0.01 (−0.02 to −0.01)	0.003	−0.01 (−0.01 to 0.01)	0.928
Model 3	−0.01 (−0.02 to −0.01)	0.012	−0.01 (−0.01 to 0.01)	0.267
**Magnesium level, mg/dl**				
Unadjusted	−0.15 (−0.31 to 0.02)	0.076	−0.07 (−0.22 to 0.07)	0.3
Model 1	−0.17 (−0.33 to −0.01)	0.049	−0.04 (−0.18 to 0.1)	0.567
Model 2	−0.15 (−0.32 to 0.01)	0.074	−0.04 (−0.18 to 0.1)	0.583
Model 3	−0.14 (−0.32 to 0.04)	0.132	−0.07 (−0.22 to 0.09)	0.4

Model 1: adjusted for sex and age.

Model 2: adjusted for body mass index and history of diabetes, cardiovascular disease, cerebral hemorrhage, stroke, and head injury in addition to the factors adjusted for in model 1.

Model 3: adjusted for calcium, phosphate, and log-transformed intact parathyroid hormone levels; urea clearance; and administration of calcimimetics, phosphate binders, and active vitamin D analogs in addition to the factors adjusted for in model 2. 25-OHD, 25-hydroxyvitamin D; A*β*, amyloid *β*; CI, confidence interval; FGF-23, fibroblast growth factor-23; NfL, neurofilament light chain.

## Discussion

In this study, we examined the association between blood biomarkers and cognitive function in patients undergoing hemodialysis and found that NfL levels showed a weak negative association with MMSE and MoCA scores, whereas the A*β*1–42/1–40 ratio showed no such association. Among the CKD-MBD–related factors, the *α*-Klotho level showed a weak positive correlation with the NfL level and A*β*1–42/1–40 ratio, whereas the 25-OHD level showed a weak negative correlation with the A*β*1–42/1–40 ratio. In addition, the FGF-23 level showed a weak positive correlation with NfL levels in some analyses. Few studies have examined the associations between NfL levels, cognitive function, and CKD-MBD–related factors in patients undergoing hemodialysis; therefore, this exploratory study contributes to filling this gap.

The median MoCA score was 25; many patients had mild cognitive impairment, and few patients showed considerably low MMSE scores. Many of the patients had a history of diabetes or cardiovascular disease, indicating a high risk of vascular dementia. CKD-MBD was managed appropriately by clinicians in accordance with the Japanese guidelines.^22^

NfL is a protein derived from neurons, predominantly expressed in myelinated axons. In various neurologic disorders, cerebrospinal fluid NfL levels rise in relation to the extent of axonal damage, and NfL released from the central nervous system can be detected at increased concentrations in the blood.^23^ For a healthy individual 65 years or older, the reported serum NfL level is approximately 10–50 pg/ml^24^; however, the population in this study exhibited higher NfL levels than their healthy age-matched counterparts. Cerebral blood flow has been documented to decrease during hemodialysis,^25^ leading to increased white matter volume. In patients undergoing hemodialysis, ultrafiltration may damage axons within the white matter, consequently elevating NfL levels. As renal function declines in patients with CKD, blood NfL levels increase by 1.3^26^–1.6^27^ times compared with those in individuals with normal renal function; however, most studies have focused on patients with only mild renal impairment.^26,27^ Previous studies on patients undergoing hemodialysis had small sample sizes and could not show a correlation between NfL and cognitive function.^10,11^ In addition, NfL levels are influenced by factors such as age, cardiovascular disease, body mass index, and head injury, which were analyzed as explanatory variables in this study.^28,29^

In this study, the A*β*1–42/1–40 ratio was not associated with cognitive function. A previous study revealed that the A*β*1–42/1–40 ratio is a highly sensitive marker for cognitive dysfunction in patients undergoing hemodialysis.^9^ However, the population of that study was younger and had a lower prevalence of diabetes than our study population, suggesting a low cardiovascular risk. Therefore, the amyloid cascade was considered to play a more prominent role in the physiologic mechanisms leading to cognitive decline in those populations than in our study population.

Vascular cognitive dysfunction may have significantly contributed to cognitive decline in our study cohort. NfL levels have been reported to increase under some conditions associated with cognitive decline, such as Alzheimer's disease, frontotemporal dementia, dementia with Lewy bodies, and vascular dementia.^30^ Therefore, identifying the exact mechanism underlying cognitive decline and NfL elevation in this study was challenging. However, given that the A*β*1–42/A*β*1–40 ratio did not correlate with cognitive function, coupled with the high cardiovascular risk of the patients in the cohort, we assumed that vascular cognitive dysfunction likely played a significant role in cognitive decline. Our findings suggest that the NfL level is more closely associated with cognitive dysfunction than the A*β*1–42/A*β*1–40 ratio in this population.

Given the exploratory nature of the analyses and modest effect sizes observed, our findings should be interpreted with caution. The Spearman correlation coefficients between NfL and global cognitive scores were small (ρ=−0.13 for MoCA and ρ=−0.11 for MMSE); therefore, these crude associations should be interpreted carefully.

High NfL levels in our cohort should be interpreted within the pathophysiologic context of CKD and hemodialysis. NfL is partly cleared through the kidneys, and patients with advanced kidney failure exhibit elevated circulating NfL levels irrespective of overt neurodegeneration. Accordingly, the relatively high NfL levels observed in our cohort may be expected even among individuals undergoing hemodialysis with only mild-to-moderate cognitive impairment. This interpretation is consistent with recent reports demonstrating that blood-based Alzheimer's disease biomarkers, including NfL, decrease after kidney transplantation as renal clearance improves.^31^

In addition, residual renal function was not assessed in this study; therefore, the degree to which impaired renal clearance contributed to elevated biomarker levels could not be fully evaluated. Because even minimal residual renal function may influence circulating NfL and amyloid levels, future studies should examine these biomarkers across CKD stages and incorporate precise measurements of renal clearance.

The sensitivity of ELISA should be considered when interpreting our findings. Although we used highly sensitive kits to evaluate blood biomarker levels in this study, we cannot entirely exclude the possibility that the measurement precision of the ELISA kits was insufficient to detect minimal differences, which could have affected the A*β*1–42/A*β*1–40 ratio. Indeed, in studies involving A*β* and NfL, high-precision techniques such as single-molecule array methods and mass spectrometry are often used.^28,32,33^ However, these advanced methods are costly and require expertise, making them impractical as routine screening tools in clinical practice.^34^ Conversely, immunologic methods are more accessible in clinical settings. Our findings indicate the potential for integrating blood biomarker testing into the clinical management of patients undergoing hemodialysis.

Several limitations should be acknowledged. First, cognitive function was assessed using the MoCA and MMSE, which are screening tools with limited sensitivity compared with comprehensive neuropsychologic batteries. The distribution of cognitive scores in our cohort was relatively narrow, with fewer individuals exhibiting severe impairment than are typically observed in hemodialysis populations; this restricted range may have attenuated the strength of the observed associations. Second, cognitive testing was conducted during hemodialysis sessions, and intradialytic hemodynamic fluctuations, symptoms, or fatigue may have influenced performance. Third, blood biomarkers were measured in serum rather than plasma, the latter being the preferred matrix for NfL and amyloid assays; thus, matrix-related differences should be considered when interpreting absolute concentrations. Fourth, the cross-sectional design precludes causal inference, and the exploratory nature of the analyses necessitates cautious interpretation of the modest effect sizes observed. Fifth, cerebrospinal fluid biomarker concentrations and brain imaging data were not available, limiting the ability to directly relate serum biomarker levels to central pathology. Sixth, tau protein levels could not be accurately measured using ELISA and were therefore not included. Finally, dementia was not formally diagnosed, although we used two different types of cognitive tests to enhance the reliability of the cognitive function assessment.

As an exploratory analysis, this study provides initial evidence suggesting a possible link between blood NfL levels and cognitive impairment in patients undergoing hemodialysis. These findings are intended to promote the generation of hypotheses rather than to confirm causal relationships. To deepen our understanding of the mechanisms and move closer to clinical application, future research efforts should include prospective studies that incorporate tau protein measurement and neuroimaging modalities, such as diffusion tensor image analysis along the perivascular space index and glymphatic clearance assessment.

In conclusion, this exploratory study showed that the NfL level was weakly negatively associated with cognitive function in patients undergoing hemodialysis.

## Data Availability

Original data generated for the study will be made available on reasonable request to the corresponding author. Data Type: Observational Data. Reason for Restricted Access: The data that support the findings of this study are not publicly available because they contain information that could compromise the privacy of research participants. However, they are available from the corresponding author on reasonable request.

## Declarative Statements

This study includes clinical experimentation and received Institutional Review Board or Ethics Committee approval. All patients provided written informed consent. This study includes clinical experimentation and complies with the Declaration of Helsinki.
